# The pervasive socio-economic and psychological burden of onchocerciasis in endemic communities of Bo District, Sierra Leone: an explanatory sequential mixed-methods study

**DOI:** 10.1186/s12889-026-28720-3

**Published:** 2026-07-27

**Authors:** Augustine Bob Moseray, Alhaji Brima Gogra, Rashid Ansumana

**Affiliations:** 1 Department of Public Health, School of Medical Health Sciences, Njala University, Bo Campus, Bo, Sierra Leone; 2 Ernest Bai Koroma University of Science and Technology, Port Loko Campus, Port Loko, Sierra Leone; 3 School of Medical Health Sciences, Njala University, Bo Campus, Bo, Sierra Leone; 4 Department of Public Health, School of Community Health Sciences, Njala University, Bo Campus, Bo, Sierra Leone

**Keywords:** Onchocerciasis, Neglected tropical diseases, Socio-economic burden, Stigma, Mental health, Mass drug administration, Sierra Leone, Mixed-methods

## Abstract

**Background:**

Onchocerciasis (river blindness) persists in Bo District, Sierra Leone, despite decades of ivermectin-based mass drug administration (MDA). Programmatic efforts have prioritized parasitological and ocular outcomes, so the socio-economic and psychological burden of the disease within affected communities remains poorly quantified. We aimed to determine the magnitude, structure and distribution of this non-clinical burden.

**Methods:**

We used an explanatory sequential mixed-methods design (QUAN → qual). In the quantitative phase, 1,500 adults across five endemic chiefdoms of Bo District (Bagbwe, Baoma, Gbo, Selenga, Tikonko) were surveyed using multistage cluster sampling. Socio-economic impact (range 0–2) and psychological impact (range 0–3) were each measured with a formative composite index summing binary items on economic hardship, education/employment obstacles, emotional well-being, social stigma, and self-esteem. Instruments were forward- and back-translated into Mende, pilot-tested (Cronbach’s α = 0.76–0.81), and administered on Android tablets using ODK Collect. We used chi-square tests, one-way ANOVA, independent-samples t-tests and Spearman correlation in JASP (v0.18). Eleven key informant interviews and eight focus group discussions (five participants each, *n* = 40) were analysed thematically to explain the quantitative findings. Ethical approval was granted by the Njala University Institutional Review Board (NUIRB) on 14 April 2024.

**Results:**

The burden was extensive: 71.7% (95% CI 69.4–74.0) of respondents reported economic hardship and 95.8% (95% CI 94.7–96.7) scored at least one psychological impact. The socio-economic and psychological indices were statistically independent (ρ = .026, *p* = .311) and did not differ significantly across chiefdoms (*F*(4,1495) = 1.91, *p* = .106), sexes (t(1498) = 0.26, *p* = .796), or age groups (*F*(2,1497) = 0.80, *p* = .451). Qualitative themes described a productivity-poverty cycle, stigma experienced as “social death,” and an unrecognized gendered caregiving load.

**Conclusions:**

Onchocerciasis imposes a pervasive, uniformly distributed dual burden in Bo District that is invisible to parasitological surveillance. Morbidity-management, stigma-reduction and psychosocial support should be delivered population-wide and integrated into primary healthcare and MDA delivery rather than targeted narrowly at demographic risk groups.

## Introduction

Onchocerciasis, or river blindness, is a chronic filarial disease caused by the parasitic worm *Onchocerca volvulus* and transmitted through the bites of *Simulium* blackflies that breed in fast-flowing rivers [6, 19]. Sub-Saharan Africa carries the overwhelming majority of the global burden, and Sierra Leone has historically been among the most heavily affected countries, with the riverine ecology of districts such as Bo sustaining intense vector breeding [11, 20]. Two decades of community-directed treatment with ivermectin have substantially reduced infection intensity in Sierra Leone, yet transmission has not been interrupted and morbidity persists in endemic communities [7, 9, 10].

The public-health framing of onchocerciasis has traditionally been dominated by its ocular and dermatological sequelae and by parasitological indicators such as microfilarial load and therapeutic coverage [2]. This biomedical lens, while essential for elimination mapping, has tended to obscure the broader welfare consequences of the disease. A growing body of evidence reframes onchocerciasis, and neglected tropical diseases (NTDs) more generally, as a comprehensive assault on livelihoods and well-being: the visible, chronic and disfiguring nature of advanced disease generates productivity loss, catastrophic health expenditure, profound stigma and psychological distress that extend well beyond clinical morbidity [1, 12, 13, 18]. Productivity losses attributable to NTDs eligible for preventive chemotherapy are substantial yet routinely omitted from disease-burden accounting [13], and the mental-health consequences of NTDs of the skin and eye are increasingly recognized as a major, under-measured component of total burden [1, 14, 17].

Despite this conceptual shift, the socio-economic and psychological burden of onchocerciasis in Sierra Leone has rarely been measured directly, and almost never at a scale that permits comparison across communities and socio-demographic strata. Two questions of direct programmatic relevance remain open. First, what is the magnitude and structure of the non-clinical burden is it concentrated in a vulnerable minority, or is it a generalized community condition? Second, is the burden patterned by geography or demography in ways that would justify targeted intervention, or is it uniformly distributed? Answering these questions matters because the morbidity-management and disability-prevention (MMDP) agenda the second pillar of NTD programs alongside MDA requires an accurate picture of who is affected and how, in order to allocate scarce resources [15, 16].

This study addresses that gap. Using an explanatory sequential mixed-methods design among 1,500 adults across five endemic chiefdoms of Bo District, we quantify the socio-economic and psychological impacts of onchocerciasis, test whether they vary by chiefdom and socio-demographic group, examine the relationship between the two burden dimensions, and draw on qualitative accounts to explain the observed patterns. The analysis is organized by the integrated Health Belief Model–Socio-Ecological Model (HBM–SEM) framework, which situates individual experience within community and structural context [16, 23].

## Methods

### Study design

**Fig. 1:**
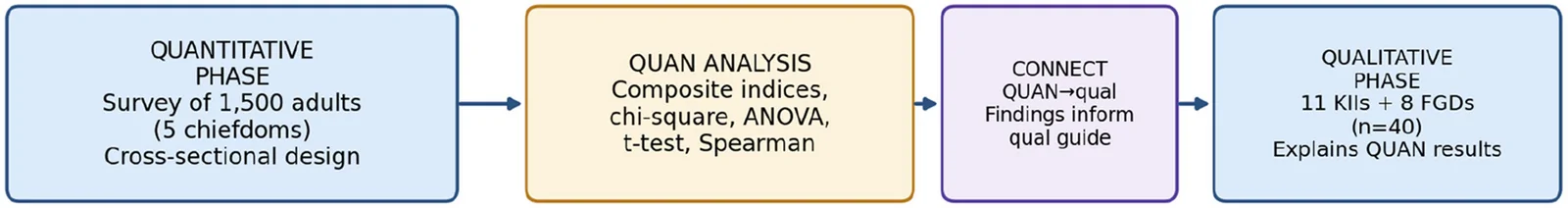
Schematic of the explanatory sequential mixed-methods design (QUAN → qual) used in this study. The quantitative survey (*n* = 1,500) was analysed first; findings were used to develop the qualitative interview/FGD guide, and the qualitative phase (11 key informant interviews; 8 focus group discussions, *n* = 40) was then used to explain and contextualise the quantitative results

This study employed an explanatory sequential mixed-methods design (*QUAN→ qual*). The integration, execution, and reporting of the mixed-methods components were guided by the Good Reporting of a Mixed Methods Study framework to ensure a transparent and comprehensive presentation of both the quantitative and qualitative strands [5, 21] (Fig. 1).

#### Study area and population

The study was conducted in Bo District, Southern Province, Sierra Leone. The district has a tropical monsoon climate and an extensive hydrological network, including the Sewa River, that creates abundant *Simulium damnosum* breeding habitat and underpins onchocerciasis endemicity [22, 24]. The economy is predominantly agrarian and riverine, and a majority of rural households live below the national poverty line. 26 rural villages across five endemic chiefdoms (Bagbwe, Baoma, Gbo, Selenga and Tikonko) were selected in consultation with the Bo District Health Management Team on the basis of documented endemicity, MDA history, treatment-coverage challenges and ecological diversity. The survey population comprised all permanently resident adults aged 18 years and older.

#### Sample size and sampling

Sample size was based on a single-proportion formula (*p* = 0.5, d = 0.05, 95% confidence), adjusted for a design effect of 1.5 and 10% non-response, yielding a minimum of 640 [3, 4]. To permit adequately powered chiefdom-level disaggregation (≥ 300 respondents per chiefdom to detect a 15-percentage-point difference at 80% power), the target was inflated to 1,500. A multistage cluster design was used: chiefdoms were purposively selected (stage 1), villages were selected stratified by ecological and socio-economic characteristics (stage 2), households were sampled systematically (stage 3), and one eligible adult per household was selected using the Kish grid (stage 4).

#### Measures

We operationalized socio-economic impact with two closed items: economic hardship and educational/employment obstacles. These items formed a socio-economic impact index (range: 0–2). We operationalized psychological impact using three closed-ended binary items: emotional well-being (assessing frequent feelings of anxiety or sadness related to the condition) social stigma and isolation (perceived or enacted exclusion from community activities), and self-esteem (self-reported loss of dignity or worth). These items were aggregated into a psychological impact index (range: 0–3) by summing the positive responses.

The selection of these indicators was informed by established research on neglected tropical diseases, which highlights that the ‘hidden’ burden of onchocerciasis often manifests through social disqualification and significant psychological distress [12, 23]. Because these items represent different qualitative dimensions of the psychological experience where an individual might experience stigma without a loss of self-esteem, or vice-versa we employed a formative measurement approach [21]. In such models, the indicators collectively define the construct, and an increase in any one item represents an increase in the overall burden [5].

This approach is consistent with recent qualitative evidence from similar endemic settings, where patients and program managers identified stigma and emotional despair as central to the onchocerciasis experience [18]. To ensure reliability in the local context of Bo District, the items were pilot-tested after forward- and back-translation into Mende, yielding a Cronbach’s *α* of 0.81, which indicates strong internal consistency for this index We recorded specific livelihood impacts, economic repercussions, community-level impacts, and healthcare access obstacles using supplementary single items. We forward- and back-translated instruments into Mende. An expert panel content-validated them. We also cognitively tested and piloted the instruments (Cronbach’s α = 0.76 for knowledge, 0.81 for stigma,test–retest intraclass correlation: 0.79–0.85). We collected data on Android tablets using Open Data Kit Collect (ODK Collect).

#### Qualitative phase

We conducted 11 key informant interviews in Mende with community drug distributors, community health officers, village chiefs, a traditional healer, and the district NTD focal person. To ensure transparency and rigor in the design, execution, and reporting of this phase, we adhered to the Consolidated Criteria for Reporting Qualitative Research guidelines. Interviews focused on the social and economic drivers of disease burden and were analyzed using a thematic approach to explain the initial quantitative. We also conducted eight focus group discussions (FGDs) with 40 community members (five participants per group), stratified by sex and accessibility. The number and size of groups were set a priori following recommended practice for FGDs on sensitive health topics in rural settings, where smaller groups of 5–8 participants facilitate open discussion of stigma-related experiences; this was confirmed adequate when no substantively new themes emerged in the final two groups, indicating thematic saturation. These were audio-recorded, transcribed, translated, and analyzed in MAXQDA 2022 using a hybrid deductive–inductive thematic approach, informed by the Health Belief Model–Social Ecological Model (HBM–SEM) framework. Analyst triangulation, member checking, and back-translation of 30% of transcripts clearly enhanced credibility.

#### Statistical analysis

We analyzed data in JASP (v0.18). We summarized categorical variables as frequencies and percentages. Composite indices were summarized as means and standard deviations. We tested associations between categorical impacts and chiefdom, sex, and education using the Pearson chi-square test. To minimize confounding from external drivers of socio-economic distress such as general rural poverty or localized industrial activities like artisanal mining we first confirmed the demographic and socio-economic comparability of the five chiefdoms [8]. To ensure the robustness of our cross-chiefdom comparisons, we verified that the five study sites were demographically comparable in terms of education level, age distribution, and household size. This mitigated demographic confounding, providing a stable background against which the impacts of onchocerciasis could be assessed. While we recognize the inherent geographic and economic heterogeneities of the district, this comparability allowed us to test whether the non-clinical burden of the disease varied by location or remained uniform across these different social strata [15, 22]. We applied Cramér’s V (or φ for 2 × 2 tables) as the effect size. We tested differences in composite indices across chiefdoms via one-way ANOVA. We tested sex-based differences using independent-samples t-tests, with Mann–Whitney U providing a non-parametric check. We assessed the relationship between the two composite indices using Spearman’s rank correlation. Significance was set at *p* < 0.05.

#### Ethics

The Njala University Institutional Review Board (NUIRB) granted ethical approval on 14 April 2024. We secured additional permissions from the Bo District Health Management Team and chiefdom authorities. All participants provided voluntary informed consent. For non-literate participants, an independent community member witnessed consent documentation by thumbprint. All outputs contain no identifying information.

## Results

### Sample characteristics

We conducted a survey of 1,500 adults. The sample exhibited a balanced sex ratio (51.0% female) and significant youth bias (54.9% aged 18–37). Educational attainment remained low (27.9% non-formal; 11.7% tertiary), with 69.3% reporting river-dependent livelihoods (farming and fishing). The five chiefdoms demonstrated demographic comparability, which minimized geographic confounding across all study sites (Table 1).

**Table 1:** Socio-demographic characteristics of respondents (*N* = 1,500)

Variable	Level	Frequency (*n*)	Percentage (%)
Sex	Female	765	51.0
	Male	735	49.0
Age (years)	18–37 y	823	54.9
	38–57 y	485	32.3
	≥ 58 y	192	12.8
Marital status	Married	843	56.2
	Single	451	30.1
	Separated	109	7.3
	Divorced	97	6.5
Education	Non-formal	418	27.9
	Primary	458	30.5
	Secondary	449	29.9
	Tertiary	175	11.7
Occupation	Farming	776	51.7
	Fishing	264	17.6
	Civil servant	199	13.3
	Other	261	17.4
Religion	Muslim	849	56.6
	Christian	651	43.4

Household size: mean 6.9 (SD ≈ 3.1). “Other” occupation combines drivers, the unemployed, housewives, students and petty traders

### Magnitude and multidimensionality of burden

**Fig. 2:**
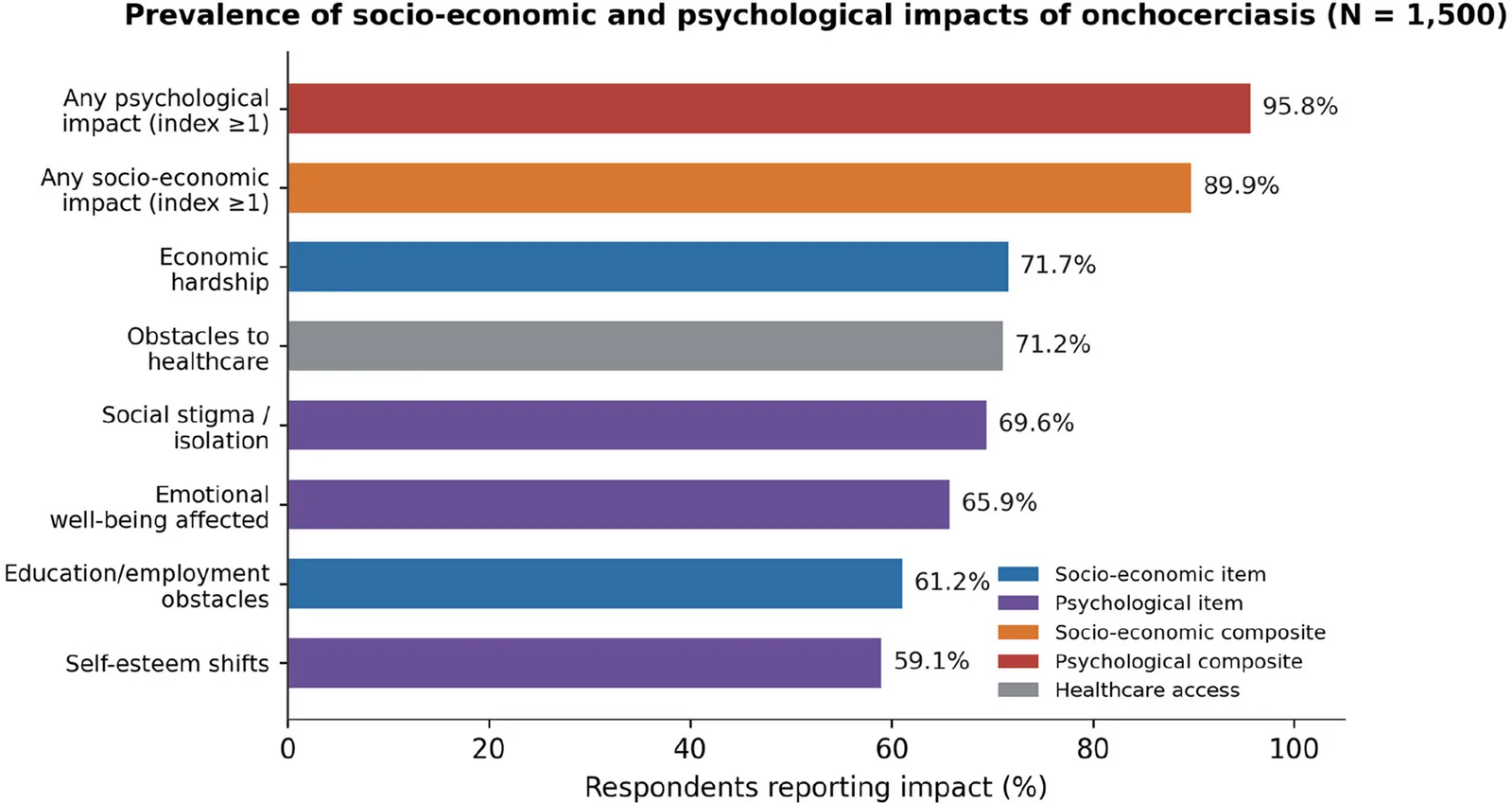
Prevalence of socio-economic and psychological impacts of onchocerciasis among 1,500 adults in five endemic chiefdoms of Bo District. Single-item indicators are shown alongside the two composite indices (proportion scoring ≥ 1)

The socio-economic and psychological consequences of onchocerciasis were pervasive (Table 2 and Fig. 2). Nearly three-quarters of respondents reported economic hardship (71.7%, 95% CI 69.4–74.0) and obstacles to education or employment (61.2%, 95% CI 58.7–63.6). The most frequently cited livelihood impacts were reduced work capacity (24.1%) and inability to perform labour-intensive jobs (23.3%), and the most enduring economic repercussions were loss of income (27.2%) and loss of livelihood (21.4%). On the psychological side, social stigma or isolation was the single most prevalent consequence (69.6%, 95% CI 67.2–71.9), ahead of impaired emotional well-being (65.9%, 95% CI 63.4–68.2) and self-esteem shifts (59.1%, 95% CI 56.6–61.5). Almost three-quarters (71.2%, 95% CI 68.9–73.4) reported obstacles to obtaining healthcare.

**Table 2:** Socio-economic and psychological impact indicators and composite indices (*N* = 1,500)

Indicator	n	% (95% CI)
Economic hardship reported	1,076	71.7 (69.4–74.0)
Obstacles to education/employment	918	61.2 (58.7–63.6)
Emotional well-being affected	988	65.9 (63.4–68.2)
Social stigma or isolation	1,044	69.6 (67.2–71.9)
Self-esteem shifts	886	59.1 (56.6–61.5)
Obstacles to obtaining healthcare	1,068	71.2 (68.9–73.4)
Socio-economic index ≥ 1 (any impact)	1,349	89.9 (88.3–91.4)
Psychological index ≥ 1 (any impact)	1,437	95.8 (94.7–96.7)

Socio-economic impact index (0–2): mean 1.33 (SD 0.65). Psychological impact index (0–3): mean 1.95 (SD 0.83). Indices are formative sums of the constituent binary items

When combined into composite indices, the burden was confirmed as pervasive: the socio-economic index averaged 1.33 of a possible 2 and the psychological index 1.95 of 3, with 89.9% (95% CI 88.3–91.4) of respondents scoring at least 1 on the socio-economic index and 95.8% (95% CI 94.7–96.7) on the psychological index (Table 2). The psychological footprint reported here is a conservative lower bound, since the survey did not measure onchocerciasis-associated epilepsy, an important but under-recognised source of neurological morbidity and stigma in endemic communities [14].

### Independence of the two burden dimensions

Although both dimensions affected the large majority of respondents, they were statistically independent: the socio-economic and psychological indices were essentially uncorrelated (Spearman ρ = 0.026, *p* = 0.311). This is most plausibly a near-ceiling effect when each dimension affects two-thirds to three-quarters of the population, little between-person variance remains for them to co-vary. We demonstrate the burden functions as a generalized community condition rather than individually graded, co-occurring impairments. We propose treating socio-economic and psychological dimensions as distinct outcomes.

### Uniformity across chiefdoms and socio-demographic groups

**Fig. 3:**
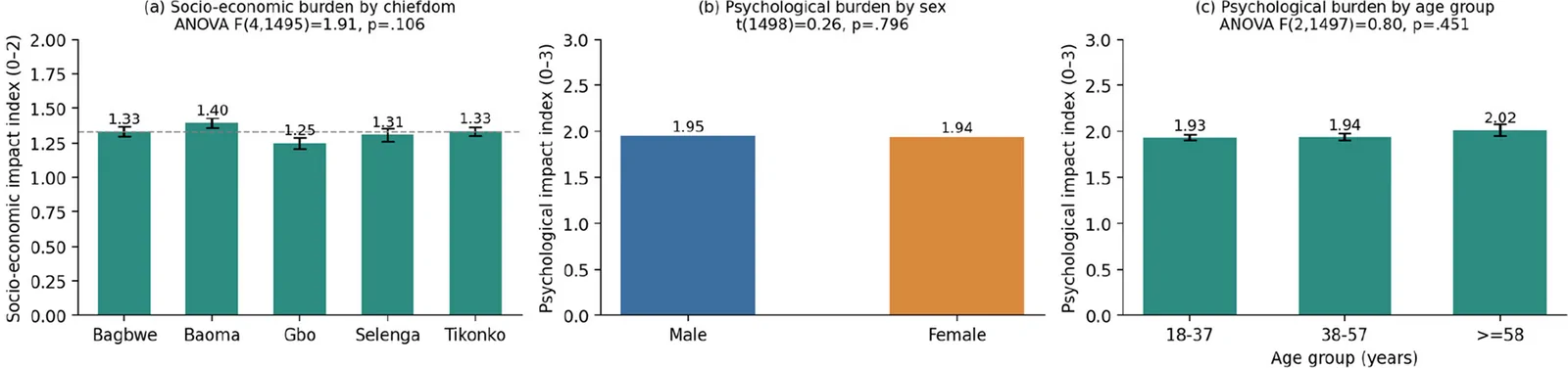
Uniformity of burden. **a** Mean socio-economic impact index by chiefdom (error bars = standard error); the dashed line marks the district mean. **b** Mean psychological impact index by sex. **c** Mean psychological impact index by age group (error bars = standard error). None of the three comparisons was statistically significant

The burden was distributed remarkably evenly across the district (Fig. 3 and Table 3). Socio-economic impact remained uniform across the five chiefdoms (*F*(4,1495) = 1.91, *p* = 0.106; Levene’s *p* = 0.222). Means spanned 1.25 (Gbo) to 1.40 (Baoma). Psychological impact showed no significant difference between men and women (t(1498) = 0.26, *p* = 0.796; means 1.95 vs 1.94). The Mann–Whitney U test confirmed this null result (*p* = 0.806). Age was analysed in three bands consistent with Table 1 (18–37, 38–57, ≥ 58 years): neither the socio-economic index (*F*(2,1497) = 0.73, *p* = 0.481) nor the psychological index (*F*(2,1497) = 0.80, *p* = 0.451) differed significantly by age group, with means for the psychological index ranging narrowly from 1.93 (18–37 y) to 2.02 (≥ 58 y). At the item level, none of the associations between specific impacts (economic hardship, stigma/isolation, self-esteem) and chiefdom, sex, education or age reached significance, and every effect size was trivial (all V ≤ 0.059). We additionally tested whether burden differed by exposure status – whether a respondent was personally diagnosed or knew someone with onchocerciasis (46.2% of the sample) versus not – as a proxy for direct versus indirect experience; neither index differed significantly by exposure status (socio-economic: t(1498) = −0.89, *p* = 0.371; psychological: t(1498) = −0.50, *p* = 0.614), suggesting the burden is felt similarly by those with and without personally proximate disease experience. A multiple linear regression of psychological impact on chiefdom, sex, education and age explained virtually no variance (*R*^2^ = 0.005; *F*(10,1489) = 0.67, *p* = 0.749). The burden of onchocerciasis is therefore not patterned by where a person lives, their sex, their education, their age, or their personal proximity to a diagnosed case.

**Table 3:** Variation of impact burden across chiefdoms and socio-demographic groups

Outcome/test	Statistic	p	Effect size
SE impact across chiefdoms (ANOVA)	*F*(4,1495) = 1.91	.106	—
PSY impact by sex (t-test)	t(1498) = 0.26	.796	—
SE impact across age groups (ANOVA)	*F*(2,1497) = 0.73	.481	—
PSY impact across age groups (ANOVA)	*F*(2,1497) = 0.80	.451	—
SE index by exposure status (t-test)	t(1498) = − 0.89	.371	—
PSY index by exposure status (t-test)	t(1498) = − 0.50	.614	—
SE–PSY index correlation (Spearman)	ρ =.026	.311	negligible
Economic hardship × chiefdom (χ2)	3.37 (4)	.498	V =.047
Stigma/isolation × chiefdom (χ2)	3.69 (4)	.450	V =.050
Self-esteem × education (χ2)	5.14 (3)	.162	V =.059
PSY impact regression (chiefdom + sex + education + age)	*R*^2^ =.005	.749	—

Age groups: 18–37, 38–57, ≥ 58 years. Exposure status: personally diagnosed or knows someone with onchocerciasis (yes/no), used as a proxy for direct versus indirect experience. Analyses demonstrate null findings (*p* ≥.05) and negligible-to-small effect sizes across all variables

*SE* socioeconomic, *PSY* psychological

### Qualitative insights of the burden

Three themes provided qualitative depth to the quantitative findings. Participants described a productivity–poverty cycle. Physical impairment reduced agricultural output. Resulting income loss precluded healthcare access. The cycle compounded. A community drug distributor in Baoma stated that a farmer kept from early-morning work by itching “will have less to sell, and then he cannot even afford to access the clinic.” Second, stigma was experienced as social death rather than mere awkwardness comprehensive exclusion from ceremonies, marriage and community life; a woman in Bagbwe recounted that her daughter’s marriage collapsed because the in-laws feared her skin “would taint their bloodline.” Third, women described an invisible, gendered caregiving burden, taking on household and care responsibilities when family members were incapacitated, without recognition or support. These accounts explain why stigma outranked even economic hardship among the psychological consequences and why the burden is felt community-wide.

## Discussion

This study was conducted at a point where parasitological indicators in Bo District have fallen substantially after two decades of mass drug administration (MDA), and it may reasonably be asked why the non-clinical burden of onchocerciasis warrants attention at this late stage of the elimination effort. We suggest the timing is precisely what makes the question important: as districts such as Bo approach the entomological and serological thresholds for stopping MDA, the socio-economic and psychological consequences of past and residual disease risk becoming invisible to the surveillance systems that drive donor and government decision-making, even though affected individuals and communities continue to live with them. Documenting that this burden remains pervasive and largely undifferentiated by geography or demography is therefore intended to support the case for sustaining morbidity-management and disability-prevention (MMDP) services through the final mile of elimination and beyond, rather than to argue against progress toward elimination itself.

Our finding of a ‘uniform’ burden across chiefdoms must be interpreted alongside recent evidence that onchocerciasis transmission in Sierra Leone remains highly focal and river-dependent [9, 19]. While the biological risk is geographically concentrated, our data suggests that the resulting socio-economic and psychological ‘ripples’ such as stigma and productivity loss diffuse more broadly across the community [23]. We also considered whether localized economic drivers, such as the artisanal mining activities in Baomahun, might independently skew the results [22].

Despite these varying economic landscapes, the lack of significant differences in impact scores across chiefdoms (*F=1.91,p=.106*) suggests that onchocerciasis-related distress is a pervasive ‘common denominator’ [12]. This implies that the ‘welfare assault’ of the disease functions as a generalized community stressor in endemic zones, overriding the specific geographic or industrial characteristics of individual chiefdoms [16, 18].

These results align with, and extend, the wider literature reframing onchocerciasis and other NTDs as assaults on welfare rather than purely clinical conditions [13, 16, 18]. The prominence of stigma echoes Weiss’s account of the social burden of NTDs and the disability mental-health nexus described by Kuper, and the productivity–poverty cycle our participants described mirrors the documented productivity losses and catastrophic health expenditure associated with NTDs in sub-Saharan Africa [12, 13, 15]. The closest comparator a qualitative study of onchocerciasis perception, impact and coping in south-east Nigeria similarly found that economic hardship and stigma dominated patients’ accounts [18]. Our contribution is to show, at scale and quantitatively, that this burden is pervasive and uniformly distributed.

The uniformity finding has direct programmatic significance. Where almost everyone is affected and no subgroup bears a disproportionate share, demographically targeted morbidity management is poorly matched to the epidemiology; a population-wide approach is indicated. The independence of the socio-economic and psychological dimensions further implies that economic-support and psychosocial-support interventions address partly different people and should be designed as distinct, parallel pathways rather than assumed to co-occur. Practically, this argues for embedding MMDP including mental-health screening, stigma reduction and community-based rehabilitation into routine primary healthcare and MDA delivery, the provision our data found in shortest supply, since 71.2% reported obstacles to obtaining care [15]. Training community drug distributors to recognise psychological distress during ivermectin delivery, and building stigma-reduction messaging into community engagement, are low-cost, feasible entry points in this resource-constrained setting.

Several limitations temper interpretation. The cross-sectional design precludes causal inference and cannot capture temporal change in burden. Impacts were self-reported and measured with closed single-response items rather than validated psychometric scales, so the composite indices are formative sums rather than latent constructs; the near-ceiling prevalence also limits variance available for subgroup contrasts, which may partly explain the uniform null results. The survey did not directly distinguish whether respondents were reporting direct personal experience of onchocerciasis or experience mediated through affected relatives, neighbours or community knowledge; as a proxy, we tested whether respondents who were personally diagnosed or knew someone with the condition (46.2% of the sample) reported greater burden than those who were not, and found no significant difference on either index (Table 3), suggesting the non-clinical burden diffuses through the community regardless of proximate exposure. This proxy is imperfect, however, since it does not itself separate self-experience from others’ experience, and disentangling the two remains an important target for future instrument design, particularly as prevalence continues to fall with MDA. Similarly, the education/employment obstacle item combined school- and work-related barriers; age-group comparisons on the composite indices showed no significant differences (Table 3), but we could not disaggregate this specific item by age within the present closed-item design. The survey did not measure onchocerciasis-associated epilepsy, so total neuropsychiatric burden is underestimated [14]. Multistage cluster sampling may modestly inflate apparent precision. Finally, the study was conducted in five purposively selected endemic chiefdoms of one district and may not generalise to non-endemic or urban settings.

## Conclusions

Onchocerciasis imposes a pervasive, multidimensional socio-economic and psychological burden on endemic communities in Bo District. This burden remains invisible to standard parasitological surveillance. While transmission is geographically focal, the resulting social and economic impact affects the community uniformly across different chiefdoms and socio-demographic groups. This uniformity holds even when accounting for localized economic drivers like artisanal mining, suggesting that the condition acts as a generalized community stressor. We propose pairing MDA with population-wide morbidity management, stigma reduction, and integrated psychosocial support services.

## Data Availability

The datasets generated and analysed during the current study are not publicly available in order to protect the confidentiality of participants from small endemic communities, where indirect identification is possible, but the de-identified quantitative dataset is available from the corresponding author (A.B.M.) on reasonable request. Qualitative interview and focus-group transcripts cannot be shared in full to preserve participant anonymity, but de-identified excerpts are available from the corresponding author on reasonable request. All requests will be reviewed in line with the ethical approval granted by the Njala University Postgraduate Review Board.
